# Soil nutrient assessment based on attribute recognition model in the Loess Plateau of China

**DOI:** 10.1186/2193-1801-2-S1-S14

**Published:** 2013-12-11

**Authors:** Feng Jiao, Zhong-Ming Wen, Shao-Shan An

**Affiliations:** Institute of Soil and Water Conservation, Northwest A&F University, Yangling, Shaanxi China; Institute of Soil and Water Conservation, Chinese Academy of Science and Ministry of Water Resource, Yangling, Shaanxi China

**Keywords:** soil fertility, attribute recognition model, entropy weight, vegetation succession

## Abstract

Soil fertility is important factors for growth and productivity of vegetation. The relationship between vegetation and soil fertility deserves attention due to its scientific importance and practical applications. However, the effects of soil fertility on vegetation development and succession are poorly documented. Here we study soil fertility in Yanhe watershed at northern Shaanxi on five different land uses, namely shrubland, farmland, natural grassland, woodland, and artificial grassland, and in soil under restoration for 5, 10, 15, 20, and 25. Attribute recognition model based on entropy weight was used to evaluate the soil fertility of typical region in the Loess Plateau of China, which contained 52 soil samples with 6 physical and chemical indexes, including soil organic matter, soil total nitrogen, total phosphorus, etc. The results show that (1) Land use has an obvious effect on soil bulk density, total porosity and capillary porosity of surface layers, but not significant in the subsurface layer; (2) SOM, N_total_, N_hydro_ and K_avail_ are the most in shrubland and woodland while P_total_ and P_avail_ in farmland, respectively; (3) Vegetation succession on eroded soil result in significant changing of soil fertility; and (4) Vegetation succession on eroded soil result in significant changing of soil fertility.

## Introduction

Soil fertility is important factors for growth and productivity of vegetation [1–3]. Vegetation structure, soil moisture and nutrients have very close relationship. Different soil nutrients affect vegetation community the size of the biomass, species composition and diversity [4]. Soil nitrogen determines the productivity, biodiversity and species invasive capacity of vegetation communities [5–7]. Phosphorus is a restrictive factor in a variety of soil types, and determines the size of vegetation productivity and change of species composition [8–12]. Potassium also affects community biomass [10] and state of vegetation water, and help to overcome soil moisture stress [13]. So, in vegetation restoration and reconstruction, it is should be considered that soil properties of abandoned farmland to assure that the ideal and realistic restoration goals [4]. However, over exploitation of existing vegetation further aggravates the problem of land degradation and supply of fuel and fodder in this area, and reduced nutrients retention [14]. Deterioration of soil fertility is important in vegetation restoration, especially for converting agricultural land to reforested plantations or grassland. This topic is also important in estimating the role of natural vegetation recovery in soil rehabilitation of the Loess Plateau, where little natural vegetation exists, helping to guide current restoration of vegetation in west China.

Study of degradation processes attracts attention to the influence of degradation on the human environment, but study of recovery processes is more important, providing recommendations for eco-environmental reconstruction or rehabilitation. Much research has been done recently on the influence on soil fertility properties of vegetation recovery or different land-use patterns [15–17]. However, changes of soil fertility properties are still under study during the long-term recovery of vegetation. Research into changes of soil fertility properties is considered necessary to understand the ecological consequences of vegetation recovery [18, 19]. In the semiarid area of the Plateau, vegetation recovery or reconstruction is always limited by shortage of fertility. There is not much literature concerned with this particular issue, especially for long-term change of soil fertility properties under natural re-vegetation in the Plateau [20]. The objective of the present study is to identify changes in soil fertility in five different land uses including shrubland, natural grassland, artificial grassland, farmland, and woodland, and changes in soil fertility after different restoration periods of plantations. We hypothesized that soil fertility properties are largely a function of secondary succession re-growth. Other important factors, such as neighboring vegetation, climate change, and altitude were not considered. The most popular natural grassland in the study area with vegetative chronosequence is also investigated to evaluate soil fertility on lands with different restoration times.

## Materials and methods

### Study area

**Figure 1:**
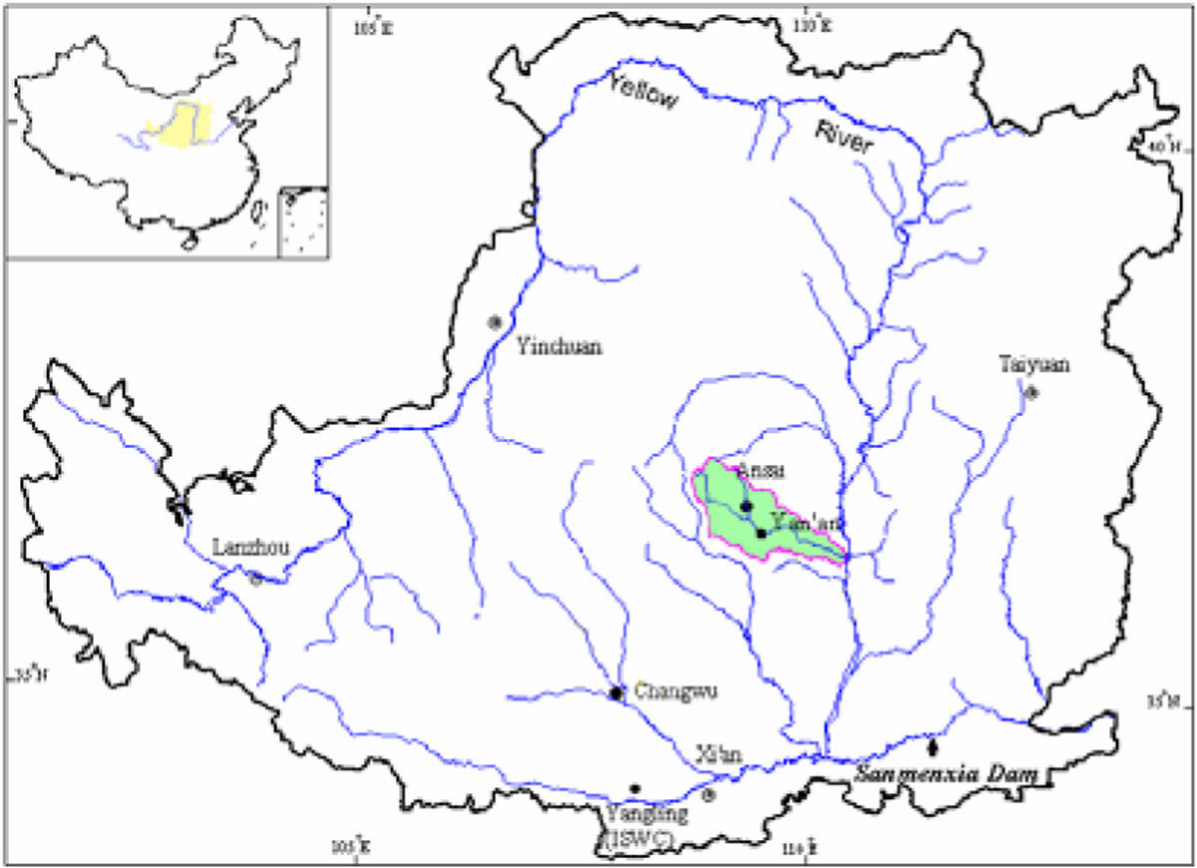
**The location of study area on the Loess Plateau**.

The study area was located in Yanhe watershed of the Loess Plateau at N 36°23′-37°17′and E 108°45′-110°28′ in northern Shaanxi Province and had 25 years of comprehensive management because one ecology station was founded in this area (Figure 1). Of the area, 287 km of length, 7687 km2 of the total area; 90% is hilly, 3% is villages, rivers, and lakes; and only 7% is considered suitable for intensive agriculture. The study area has a semi-arid climate characterized by heavy seasonal rainfall with periodic local flooding and drought; the average annual rainfall at the experimental site is 497 mm (1970-2000, CV22%) with distinct wet and dry seasons. The rainy season starts in July and continues up to October; the August rainfall accounts for 23% of the annual rainfall. The annual reference evapotranspiration is approximately 1000 mm. Most of the lands are located at 900-1500 m altitude and are closely dissected and sharp-edged with steep and very steep slopes (the slopes are deep, 40%). The topography, soil type, soil and land-use patterns of Yanhe watershed are very typical in the Loess Plateau, Land-use types including: sloping land, terraces, orchards, woodland, shrubland, natural grassland, wasteland and other types [21].

### Study approach and sampling design

The chronosequence method was used because of the existence of similar conversion history in this area. The management was similar, with known periods of cultivation climate, topography, and soil type. Soil samples were collected in August 2006. Soil samples were taken at 0-20 cm depths. Composite samples of about 1 kg were collected with 5 replicates at each sampling plot and then air-dried and sieved through 1 mm sieve. All measurements were made at the State Key Laboratory of Soil Erosion and Dryland Farming on the Loess Plateau, China.

The analytical methods for the soil samples were the international standard methods as adopted and published by the Institute of Soil Science, Chinese Academy of Sciences (1978). Soil organic matter (SOM) was determined on the basis of oxidation with potassium dichromate in a heated oil bath. Total nitrogen (Ntotal) was measured according to the semi micro Kjeldahl method and hydrolysable nitrogen (Nhydro) by means of the Alkali diffusion method. Total phosphorus (Ptotal) was digested with perchloric acid and sulfuric acid and determined using colorimetry. Total potassium (Ktotal) was digested with hydrofluoric acid and perchloric acid. Available phosphorus (Pavail) was extracted with sodium bicarbonate and measured with colorimetry. Available potassium (Kavail) in soil was extracted with ammonium acetate.

A common approach in studies of soil rehabilitation in relation to vegetative cover is to monitor plant and soil changes occurring along a vegetative chronosequence developed on similar soils under similar climatic conditions [22]. This chronological approach has been widely used in applied ecosystem research [23] and is considered retrospective research because existing conditions were compared with known original conditions and treatments. The retrospective approach was adapted in this study because of the availability of closely located vegetation community established 5, 10, 15, 20 and 25 years ago on eroded soils with similar properties. These vegetation communities therefore provide a time gradient of grass occupancy on similar sites. Changes in soil properties can be measured by comparing sites of different ages. Five age series (5-, 10-, 15-, 20- and 25-year-old vegetation community) were found in the adjacent sites of the study area, which have undergone light livestock grazing in recent years. Within each community (5, 10, 15, 20 and 25), five sites were selected as sampling (five replicates). Also, five nonvegetated lands in the vicinity of the planted sites (farmland) were chosen as a control for the chronosequence.

### Calculation of soil samples attribute measure and data analysis

Soil samples attribute measure is its status value in soil. It is used that the second national soil survey classification of soil nutrient standards for the evaluation criteria, and a standard matrix was built up based on the evaluation criteria. Soil sample analysis of variance (ANOVA) and correlation were carried out using the SPSS11.0 procedures for sites in different succession stages. Duncan's test (at p < 0.05) was used to compare means of soil variables when the results of ANOVA were significant at p < 0.05.

## Results and discussions

### Soil fertility in different land uses

Table 1 shows that, in different land use, changing SOM, Ntotal, Ptotal, Nhydro, Pavail and Kavail were significant in the 0-20 cm layer at p < 0.05. Woodland contains the highest all soil fertility indexes except Pavail. Farmland contains the highest Ptotal (0.57%) and Pavail (2.06 mg/kg), and has a higher Kavail (60.2 mg/kg). Natural grassland and Shrubland contain the highest Kavail (70.0 mg/kg), and have higher SOM (8.11%) and Ntotal (0.46%), respectively. While artificial grassland has a lower contain in all soil fertility indexes.

**Table 1:** Means and coefficient of variations of soil nutrient in different land-use patterns

Land use	SOM (%)	N_total_(%)	P_total_(%)	N_hydro_(mg/kg)	P_avail_(mg/kg)	K_avail_(mg/kg)
Farmland	6.87^bc^(0.30)	0.44^bc^(0.22)	0.57^a^(0.05)	26.5^cd^(0.25)	2.06^a^(0.43)	60.2^ab^(0.37)
Artificial grassland	6.17^c^(0.32)	0.35^c^(0.22)	0.52^b^(0.03)	25.0^d^(0.12)	0.87^b^(0.64)	38.4^b^(0.15)
Natural grassland	8.11^b^(0.24)	0.46^b^(0.32)	0.52^b^(0.12)	34.8^b^(0.23)	0.88^b^(0.13)	70.0^a^ (0.27)
Woodland	10.25^a^(0.19)	0.61^a^(0.15)	0.57^a^(0.06)	44.4^a^(0.19)	1.01^b^(0.37)	85.3^a^(0.47)
Shrubland	7.08^bc^(0.27)	0.46^b^(0.27)	0.53^ab^(0.08)	32.6^bc^(0.27)	0.72^b^(0.34)	68.0^a^(0.39)
Sig. of ANOVA	0.001	0.000	0.031	0.000	0.000	0.020

Means with the same letter in the same row are not significantly different at the 0.05 level (LSD).

Data in the parentheses are coefficient of variation.

### Weight of soil fertility index in different land uses

Table 2 shows that, in different land use, changing weight of soil fertility index were significant in the 0-20 cm layer at p < 0.05. In the 0-20 cm layer, weight of Nhydro was the highest in farmland, followed by SOM, Kavail, Pavail, Ntotal and Ptotal. Weight of SOM was the highest in natural grassland, followed by Nhydro, Ntotal, Pavail, Kavail and Ptotal. Compared with that in farmland and natural grassland, weight of Kavail was the highest in artificial grassland, and weight of Nhydro and Pavail was the highest in Woodland, and weight of Ptotal was the highest in Shrubland. In comparison, SOM, Nhydro, Pavail and Kavail played an important role in different land use.

**Table 2:** Weight of soil nutrient index in different land use patterns

Land use	SOM	N_total_	P_total_	N_hydro_	P_avail_	K_avail_	Sig. of ANOVA
Farmland	19.18^b^	14.37^e^	13.77^f^	20.48^a^	14.92^d^	17.29^c^	0.000
Artificial grassland	17.64^b^	16.75^bc^	15.92^cd^	15.21^de^	15.33^de^	19.15^a^	0.000
Natural grassland	21.09^a^	17.87^c^	11.53^f^	19.17^b^	15.72^d^	14.62^e^	0.000
Woodland	13.88^c^	13.64^c^	15.89^bc^	19.59^a^	19.13^a^	17.87^ab^	0.000
Shrubland	15.62^c^	15.66^c^	19.70^a^	18.57^b^	14.87^c^	15.57^c^	0.000

Means with the same letter in the same row are not significantly different at the 0.05 level (LSD).

Data in the parentheses are coefficient of variation.

### Soil fertility in different restoration years

Five replicated soil samples were collected from five sites with the same restoration time of 5, 10, 15, 20 and 25 years, respectively. Also, five nonvegetated lands in the vicinity of the planted sites (farmland) were chosen as controls for the chronosequence. After 5, 10, 15, 20 and 25 years of restoration, SOM, Ntotal, Nhydro, Pavail and Kavail were significant in the 0-20 cm layer at p < 0.05 and except Ptotal, which was not significant at p > 0.05. Generally, vegetation succession resulted in a change of soil fertility parameters in the eroded soils, significant decreases of soil fertility parameters (p < 0.05) took place from beginning to 15-years of restoration, and significant increases (p < 0.05) from 15-years to 20-years of restoration, and significant decreases (p < 0.05) from 20-years to 25-years of restoration. The statistical results showed that soil fertility and vegetation succession had significant interactions with all soil fertility parameters except Ptotal (Table 3).

**Table 3:** Means and standard deviations of soil nutrient in different restoration years

Restoration time	SOM(%)	N_total_(%)	P_total_(%)	N_hydro_(mg/kg)	P_avail_(mg/kg)	K_avail_(mg/kg)
25years	7.33^de^(1.31)	0.43^de^(0.07)	0.54(0.07)	29.3^d^(4.29)	0.70^c^(0.16)	64.9^c^(7.79)
20years	8.02^cde^(1.47)	0.50^cde^(0.15)	0.54(0.06)	31.9^cd^(7.05)	0.67^c^(0.10)	66.0^c^(10.2)
15years	5.93^e^(0.24)	0.36^e^(0.08)	0.54(0.06)	23.2^d^(2.68)	0.72^c^(0.10)	61.2^c^(17.2)
10years	5.53^e^(0.41)	0.37^e^(0.07)	0.54(0.04)	23.3^d^(4.62)	0.83^bc^(0.18)	52.8^c^(20.5)
5years	6.62^de^(0.61)	0.43^de^(0.10)	0.53(0.03)	26.8^d^(5.99)	0.98^bc^(0.17)	54.3^c^(27.4)
0years	6.87^de^(0.30)	0.44^de^(0.22)	0.57(0.05)	26.5^d^(0.25)	2.06^a^(0.43)	60.2^c^(0.37)
Sig. of ANOVA	0.000	0.000	0.627	0.000	0.000	0.003

Means with the same letter in the same row are not significantly different at the 0.05 level (LSD).

Data in the parentheses are standard deviations.

### Weight of soil fertility index in different restoration years

**Figure 2:**
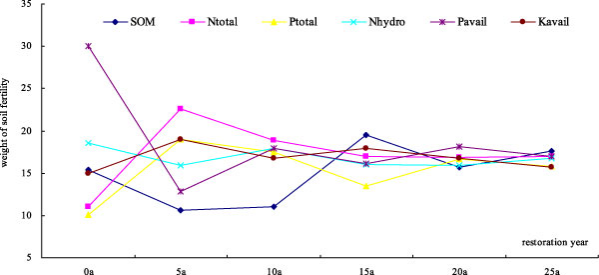
**Weight of soil fertility in different restoration years**.

Figure 2 shows that, in different restoration years, changing weight of soil fertility index was significant in the 0-20 cm layer at p < 0.05. Abandoned early, the greater the weight and so are as follows: Pavail > Nhydro > SOM > Kavail > Ntotal > Ptotal. The difference between weights of soil fertility was significant at p < 0.05, the largest up 66.28% (Figure 2). With the increase of abandoned years, the difference between weights of soil fertility was reducing gradually. The largest differences between weights of soil fertility were 52.94%, 41.84%, 30.97%, 23.04% and 11.01% in 5, 10, 15, 20 and 25 years, respectively (Figure 2). The statistical results showed that: With the increase of abandoned years, affection of soil fertility on vegetation succession came into line gradually (Figure 2).

## Conclusions

Land use has an obvious effect on Soil fertility of surface layer, but not significant in the subsurface layer. Shrubland has higher soil fertility than other land uses. In most cases, table land has low levels of soil fertility, but after long period of cultivation, the land degrades year by year. Our results indicate that the establishment and development of vegetation succession on eroded soil result in significant changing of soil fertility. With increased plantation age, it is possible to recover soil fertility to a certain degree, and affection of soil fertility on vegetation succession came into line gradually.
